# Phase Formation, Mechanical Strength, and Bioactive Properties of Lithium Disilicate Glass–Ceramics with Different Al_2_O_3_ Contents

**DOI:** 10.3390/ma15238283

**Published:** 2022-11-22

**Authors:** Arnon Kraipok, Teerapong Mamanee, Jetsada Ruangsuriya, Poomirat Nawarat, Wilaiwan Leenakul

**Affiliations:** 1 Department of Physics and Materials Science, Faculty of Science, Chiang Mai University, Chiang Mai 50200, Thailand; 2 Graduate School, Chiang Mai University, Chiang Mai 50200, Thailand; 3 Department of Restorative Dentistry and Periodontology, Faculty of Dentistry, Chiang Mai University, Chiang Mai 50200, Thailand; 4 Department of Biochemistry, Faculty of Medicine, Chiang Mai University, Chiang Mai 50200, Thailand; 5 Division of Industrial Materials Science, Faculty of Science and Technology, Rajamangala University of Technology Phra Nakhon, Bangkok 10800, Thailand

**Keywords:** lithium disilicate, heat treatment, glass-ceramic, crack propagation, Al_2_O_3_, crystals

## Abstract

Owing to its excellent mechanical properties and aesthetic tooth-like appearance, lithium disilicate glass–ceramic is more attractive as a crown for dental restorations. In this study, lithium disilicate glass–ceramics were prepared from SiO_2_–Li_2_O–K_2_O–P_2_O_5_–CeO_2_ glass systems with various Al_2_O_3_ contents. The mixed glass was then heat-treated at 600 °C and 800 °C for 2 h to form glass–ceramic samples. Phase formation, microstructure, mechanical properties and bioactivity were investigated. The phase formation analysis confirmed the presence of Li_2_Si_2_O_5_ in all the samples. The glass–ceramic sample with an Al_2_O_3_ content of 1 wt% showed rod-like Li_2_Si_2_O_5_ crystals that could contribute to the delay in crack propagation and demonstrated the highest mechanical properties. Surface treatment with hydrofluoric acid followed by a silane-coupling agent provided the highest micro-shear bond strength for all ceramic conditions, with no significant difference between ceramic samples. The biocompatibility tests of the material showed that Al_2_O_3_-added lithium disilicate glass–ceramic sample was bioactive, thus activating protein production and stimulating the alkaline phosphatase (ALP) activity of osteoblast-like cells.

## 1. Introduction

Glass-ceramics are made of crystalline phase and glass. They are specially formed composite that are created through high-temperature melting, shaping, and heat treatment. Due to their excellent mechanical strength, adjustable thermal expansion, chemical resistance, low dielectric loss, and other properties, glass-ceramics are currently gaining considerable attention in mechanical manufacturing, optics, electronics, aerospace, biomedical, and construction applications [1,2,3,4]. In the dental restoration field, glass-ceramics are widely employed due to their biocompatibility, ease of production, and chemical, physical, and mechanical qualities that are extremely similar to those of human enamel [5]. Various materials such as alumina, zirconia, leucite and lithium disilicate glass–ceramics have been developed [6]. Among these materials, lithium disilicate (Li_2_Si_2_O_5_) glass–ceramics, which are made of a crystalline ceramic and glass matrix, have drawn the most attention among these materials because of their excellent mechanical characteristics and distinctive translucency [7,8]. There has also been a significant increase in the number of reports on the composition, structure, phase transformations and properties of Li_2_O–SiO_2_-based glass in recent years [9,10,11,12,13,14,15,16]. In this context, this research focuses on LS glass–ceramics, which can be achieved by controlling the nucleation and crystallisation of the Li_2_O–SiO_2_ parent glass.

The effects of composition and heat treatment on LS glass–ceramics characteristics have been the subject of numerous studies. A high mechanical strength can be achieved in LS glass–ceramics by using P_2_O_5_ as a nucleating agent to form fine-grained interlocking microstructures [10]. An improved fracture strength can be achieved by increasing the radii of Rb and Cs ions in the lithium disilicate phase, which facilitates crystallisation [11]. When K_2_O, Rb_2_O and CsO were mixed, the fracture strength of the modified lithium disilicate glass-ceramics significantly increased [12]. CeO_2_ has a small effect on the Li_2_Si_2_O_5_ phase percentage. The best heat-treatment temperatures for samples containing 1.5 wt% CeO_2_ were 600 °C for 1 h and 800 °C for 1 h [13]. Lithium disilicate glass–ceramics can be improved by adding K_2_O and Al_2_O_3_, increasing their mechanical properties by up to 201 MPa [14,15]. The concentration of Al_2_O_3_ additive oxides on the phase formation and mechanical properties of lithium disilicate glass–ceramics prepared by using the conventional method was also studied by Leenakul and Kraipok [16]. The Al_2_O_3_ content affected the crystallisation temperature and phase formation. The highest mechanical strength was correlated with the rod-like crystals of Li_2_Si_2_O_5_. Despite the many studies related to the effect of Al_2_O_3_ on the mechanical properties of Li_2_O–SiO_2_ glass systems, to the best of our knowledge, there is no study of this effect on the modified Li_2_O–SiO_2_ glass systems with CeO_2_ yet.

This study aims to increase the Al_2_O_3_ concentration and find the optimal processing parameters for dental restorations. A SiO_2_–Li_2_O–K_2_O–P_2_O_5_–CeO_2_ glass system prepared by using the conventional melt-quenching method was used as the base glass to examine the effects of Al_2_O_3_ on the crystallisation behaviour, microstructure, mechanical properties, and bioactivity of lithium disilicate glass-ceramics. The thermal properties of these glass–ceramics were studied and managed using differential thermal analysis (DTA) to determine the crystallinity of the major phase. To better understand how the Al_2_O_3_ content affects the glass–ceramic properties, scanning electron microscopy (SEM) and X-ray diffraction (XRD) were used to examine the heated samples. Furthermore, the phase formation and mechanical and bioactive properties were tested to determine their suitability for dental restorations.

## 2. Materials and Methods

### 2.1. Glass and Glass-Ceramic Preparation

The lithium disilicate glass composition had the following mol% composition: 67.4 SiO_2_, 27.0 Li_2_O, 2.0 K_2_O, 2.0 P_2_O_5_, and 1.5 CeO_2_. The LS glass was then supplemented with Al_2_O_3_ in concentrations of 0.5, 1.0, and 1.5 mol%. LSA1, LSA2, and LSA3 were assigned to the glass samples, respectively. The raw materials for creating LS glass were SiO_2_, Li_2_CO_3_, K_2_CO_3_, (NH_4_)_2_HPO_4_, CeO_2_, and Al_2_O_3_ in the analytical grade from Sigma-Aldrich Pte. Ltd. (Singapore). In a high-temperature furnace, these raw materials were combined and melted in an alumina crucible for 2 h at 1450 °C. In order to create the glass specimens, the molten glass was quenched in a metal mould that had been heated to 500 °C. Afterward, the glass was annealed at 500 °C for 2 h before being cooled to room temperature. After being annealed, the glass samples were heated through a two-stage crystallisation process at 600 °C and 800 °C for 1 h while being heated at a rate of 2 °C/min in an air atmosphere and then cooled to room temperature.

### 2.2. Differential Thermal Analysis (DTA)

Analytical grade Al_2_O_3_ powder served as the reference material, and a differential thermal analyser (TG-DTA 8121, Rigaku, Japan) was used to determine the glass transition temperatures (T_g_), crystallisation temperatures (T_C_), and melting temperatures (T_m_) in an air atmosphere. The alumina crucible was heated at a rate of 10 °C/min from room temperature to 1200 °C with about 10 mg of glass specimen powder.

### 2.3. X-ray Powder Diffraction Analysis (XRD)

Cu-Kα radiation produced at 30 mA and 45 kV was used in the X-ray powder diffraction analysis using an X-ray diffractometer (XRD) (SmartLab, Rigaku, Japan) with a scanning range of 10° to 60° and a step size of 0.012°. Using JCPDS numbers from the ICDD-PDF2 database, the crystalline phases in the controlled heat-treated glass specimens were identified.

### 2.4. Field Emission Scanning Electron Microscopy (FE-SEM)

The microstructure of the glass-ceramic specimens was examined using field emission scanning electron microscopy (FE-SEM) (JSM-6335F, JEOL, Akishima, Tokyo, Japan)An ion sputtering device (JFC-1200, JEOL, Akishima, Tokyo, Japan) was used to coat the fracture surface of the specimens with gold for 10 s.

### 2.5. Mechanical test

Using Vickers hardness tests (Buehler, Karl Frank GMBH Type-38505, Lake Bluff, IL, USA) with a constant load of 1 kg and a dwell time of 15 s, microhardness was used to analyse the mechanical properties of the prepared glass-ceramics [17]. The equation below was used to determine the Vickers hardness (*HV*):

(1) $$HV=\frac{1.8544P}{d^{2}},$$

where 1.8544, *P*, and *d* denote the constant geometrical factor for the diamond pyramid, the applied load with the unit of kg, and the diagonal length of the indenter impression in µm, respectively.

There are universal strength machines that can be used for ISO 6872 [18] with a universal strength machine (AG-X plus, Shimadzu, Japan). The flexural modulus (*E*) was calculated using the following equation [19]:

(2) $$E=\frac{L^{3}m}{4bd^{3}},$$

where *L* is the support span (mm), m is the gradient (i.e., slope) of the initial straight-line portion of the load deflection curve, (N/mm), and b and d are the width of the test beam and thickness, respectively. A three-point-bending test with the fracture strength (σ) in the unit of MPa and a crosshead speed of 0.01 mm/min, and the test span was 16 mm. The following equation can be used to determine the three-point flexure:

(3) $$\sigma =\frac{3PI}{2wb^{2}}.$$

In this case, *P* stands for the breaking load in N, while *w*, *b*, and *l*, respectively, are the width, the thickness of the samples, and the test span in mm.

### 2.6. Micro-Shear Bond Strength (μSBS)

All the lithium disilicate glass-ceramic samples with various Al_2_O_3_ contents (LSA1, LSA2 and LSA3) were selected and subjected to the µSBS test for the evaluation of their bonding performance. Briefly, 20 ceramic plates (5 × 5 × 2 mm) of each ceramic condition were prepared and embedded in a metal ring using epoxy resin. After polishing with 600, 800, 1000, and 1200 grit silicon carbide paper, the specimens were randomly divided into 4 group according to 4 surface treatments: (1) no surface treatment; (2) silane coating (Monobond N, Ivoclar Vivadent, Liechtenstein); (3) hydrofluoric acid (IPS^®^ ceramic etching gel, Ivoclar Vivadent, Liechtenstein); and (4) hydrofluoric acid and silane coating. The treated ceramic surface was then cemented with 4 resin cement rods (Multilink^®^ N, Ivoclar Vivadent, Liechtenstein) by injecting resin cement into 4 plastic tubes (0.8 mm internal diameter and 0.5 mm height). After removing the tubes and storing in water for 24 h, the specimens were subjected to a µSBS test using universal testing machine (Instron^®^ 5566 a universal testing machine, Instron Engineering Corporation, Norwood, MA, USA) at the crosshead speed of 0.5 mm/min. Data were analysed using a two-way ANOVA test with Dunnett T3 post hoc test at a significant level of 0.05 (*p* < 0.05).

### 2.7. In Vitro Test for Biocompatibility of the Material

#### 2.7.1. Preparation of the Tested Materials

Briefly, 2 × 2 × 2 mm synthetic material was established prior to a 30-min UV irradiation process (257.7 nm, 40 W/cm^2^, ESCO UV-30A, Barnsley, England) on each side. The irradiated material was transferred into each well of a sterile 96-well plate for cell culture.

#### 2.7.2. Culture of Osteoblast-Like Cells

MG-63, an osteosarcoma cell line (ATCC, CRL1427) was used to test with the synthetic material. The cell was cultured in Dulbecco’s modified Eagle’s medium (DMEM) with the supplementation of a 10% fetal bovine serum (FBS), 2% N-(2-hydroxyethyl) piperazine-N’-(2-ethane sulfonic acid) (HEPES), 1% non-essential amino acid, and 1% penicillin/streptomycin until they reached 80% confluence. Then 1 × 10^4^ cells were loaded in the well containing the treated material with a volume of 100 μL and the plate was incubated in a humidified incubator under 37 °C and 5% CO_2_ for 24 h. The fresh medium was then replaced and the plate continued to be incubated for 48 and 96 h.

#### 2.7.3. Cell Attachment, Cell Viability, and Cell Proliferation by MTT Assay

Briefly, a 5 mg/mL methyl thiazolyl diphenyl-tetrazolium bromide (MTT) solution was prepared in a sterile phosphate-buffered saline (PBS) solution, pH 7.4 and added into each well at 10 μL. After that, the plate continued to be incubated for 4 h to allow the formation of the formazan crystal by viable cells. The media supernatant was then replaced with 100 μL dimethyl sulfoxide (DMSO). The absorbance at 540 nm was then recorded with a plate reader machine.

#### 2.7.4. Preparation of the Cell Lysate

The celll layer was primarily washed twice with 37 °C sterile PBS prior to the addition of 50 μL cell lysis buffer. The cells were exposed to the lysis buffer at room temperature for 15 min to completely lyse. The lysate was collected and stored in an Eppendorf pipette under −20 °C for further analyses.

#### 2.7.5. Total Protein Determination by Bradford Assay

The total protein content was analysed in 5 μL of the cell lysate by mixing with 100 μL of the working Bradford solution. The complete reaction was allowed to incubate for 15 min at room temperature. The absorbance was recorded with a plate reader at 595 nm. Like the sample, standard bovine serum albumin (BSA) was used to establish the standard curve for the deduction of total protein content in each sample.

#### 2.7.6. Alkaline Phosphatase (ALP) Activity Determination

Briefly, 20 μL of the lysate was mixed with 80 μL of 1 mg/mL *p*-nitrophenolphosphate in a diethanolamine buffer pH 9.8 for 3 h and the yellow product was determined by with a plate reader to measure the absorbance at 405 nm. Along with the *p*-nitophenol (pNP) standard, ALP activity could be elucidated and expressed in the specific activity per mg of the total protein.

#### 2.7.7. Statistical Analysis

ANOVA followed by Tukey’s pos-hoc test was used when the ANOVA result showed a significant difference. The *p*-value was set at 0.05 in all the statistical procedures.

## 3. Results and Discussion

### 3.1. Thermal Analysis

**Figure 1:**
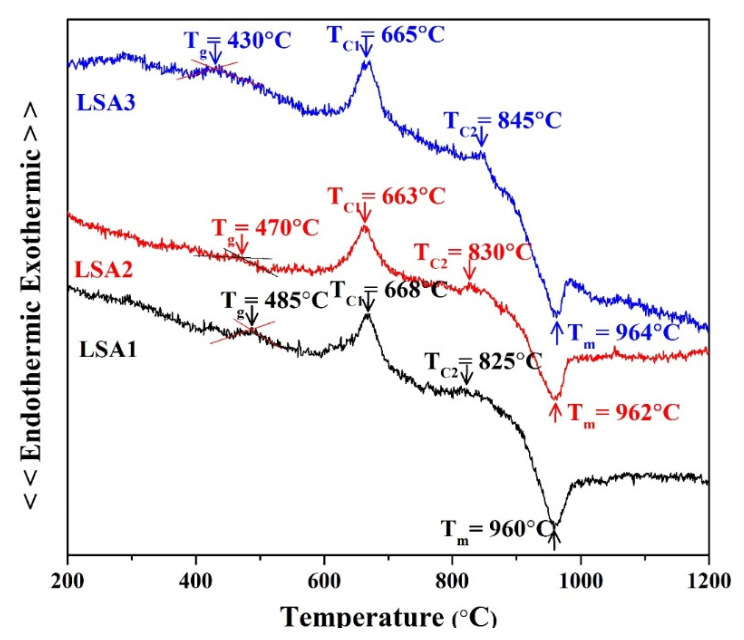
DTA curves of Li-Si glass powder with different Al_2_O_3_ contents.

Figure 1 illustrates the effect of the Al_2_O_3_ concentration on the thermal parameters of the glass specimens by performing DTA at a heating rate of 10 °C/min while maintaining an air atmosphere throughout the experiment. The letters T_g_, T_C1_, T_C2_, and T_m_ indicate the temperatures at which the glass transition, first crystallisation, second crystallisation, and melting temperature occur in the DTA curves. Exothermic peaks indicate that the glass is crystallised, whereas endothermic peaks indicate that crystalline phases dissolve within the glass matrix. In the ranges of 663–668 °C and 825–845 °C, the results for each glass sample showed two exothermic peaks. The Al_2_O_3_ content was also found to have a negligible effect on the second crystallisation and melting temperature, which corresponds to a previous study [16]. It is possible that this is because Al_2_O_3_ typically makes the glass more viscous, which is caused by the elimination of nonbridging oxygen sites [14]. In addition, T_C1_ is the temperature at which the lithium metasilicate (Li_2_SiO_3_) phase begins to crystallise. The second crystallisation temperature, T_C2_, denotes the phase transition at which the crystallised LM phase changes into the lithium disilicate (Li_2_Si_2_O_5_) phase [20,21,22]. According to the DTA results, the heat treatment condition was selected as two stages at 600 °C and 800 °C for 2 h at each temperature. It was speculated that the preliminary glass nucleation would occur at the initial stage at 600 °C and then the crystalline phase of Li_2_Si_2_O_5_ would start to occur at the final stage at 800 °C.

### 3.2. Phase Formation

**Figure 2:**
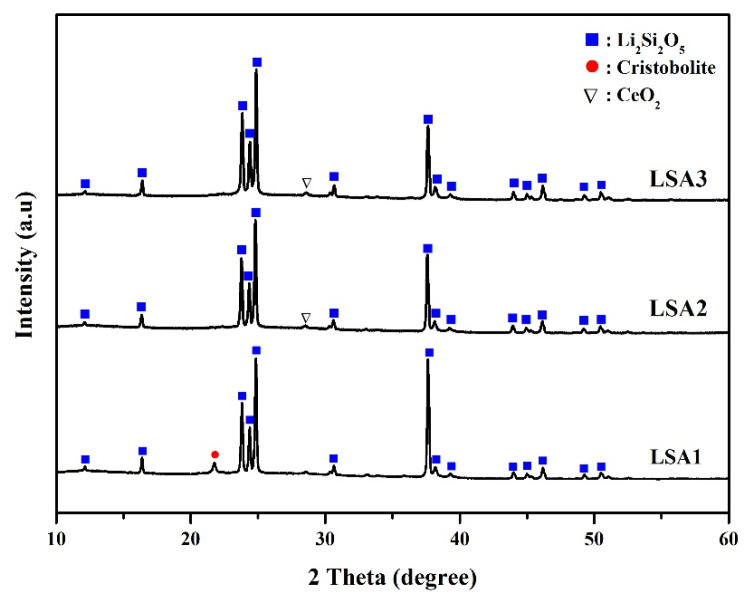
XRD analysis of glass-ceramic samples with various Al_2_O_3_ contents after heat treatment.

The phase formation of the glass-ceramic samples was investigated by forming XRD analysis, which showed the precipitating crystalline phase as a function of the various Al_2_O_3_ contents. The XRD results of all the LSA glass-ceramic samples after heat treatment at 600 °C and 800 °C for 2 h are shown in Figure 2. The XRD results revealed that Li_2_Si_2_O_5_ was the main crystalline phase in all the samples. The highest intensity of the Li_2_Si_2_O_5_ peak was obtained when an Al_2_O_3_ content of 1%mol was added, and it coexisted with a small amount of cristobalite phase. This finding is consistent with the DTA results at 800 °C; this is close to the crystalline temperature of all the glass samples, especially LSA1, which showed a T_c_ at 825 °C, while LSA2 and LSA3 were at 830 °C and 845 °C, respectively. Therefore, the crystallisation reaction of Li_2_Si_2_O_5_ was more complete, and the highest intensity peak was observed. The crystalline phase of Li_2_Si_2_O_5_ was transformed in two steps via the following reaction:

Li_2_O (glass) + SiO_2_ (glass) → Li_2_SiO_3_ (crystal), (4)

Li_2_SiO_3_ (crystal) + SiO_2_ (crystal) → Li_2_Si_2_O_5_ (crystal). (5)

Generally, in this Li_2_O–SiO_2_ glass system, the first crystalline phase of Li_2_SiO_3_ is formed by a reaction between Li_2_O and SiO_2_ (Equation (4)) at a lower temperature of 550–650 °C [22,23,24]. However, when the heat treatment temperature increased, Li_2_SiO_3_ became unstable and transformed into the more stable phase of Li_2_Si_2_O_5_, as shown in Equation (5).

The amounts of the Li_2_Si_2_O_5_ phase decreased with the increasing Al_2_O_3_ content, while the cristobalite phase disappeared and CeO_2_ started to form in the LSA2 samples. Moreover, the CeO_2_ phase remained almost unchanged with a further increase in the Al_2_O_3_ content in LSA3. This may also explain why the cristobalite phase is metastable and cannot be retained at higher Al_2_O_3_ contents. However, the reaction that transformed to Li_2_Si_2_O_5_ was incomplete and showed impurities in the CeO_2_ phase in the LSA2 and LSA3 samples. This may be attribute to the addition of Al_2_O_3_ leading to a decrease in the crystal growth velocity [25], so when the Al_2_O_3_ content exceeded the solution limit (LSA2 and LSA3), a foreign CeO_2_ phase was residual in the glass-ceramic samples, which led to the impurity of the materials.

### 3.3. SEM Analysis

**Figure 3:**
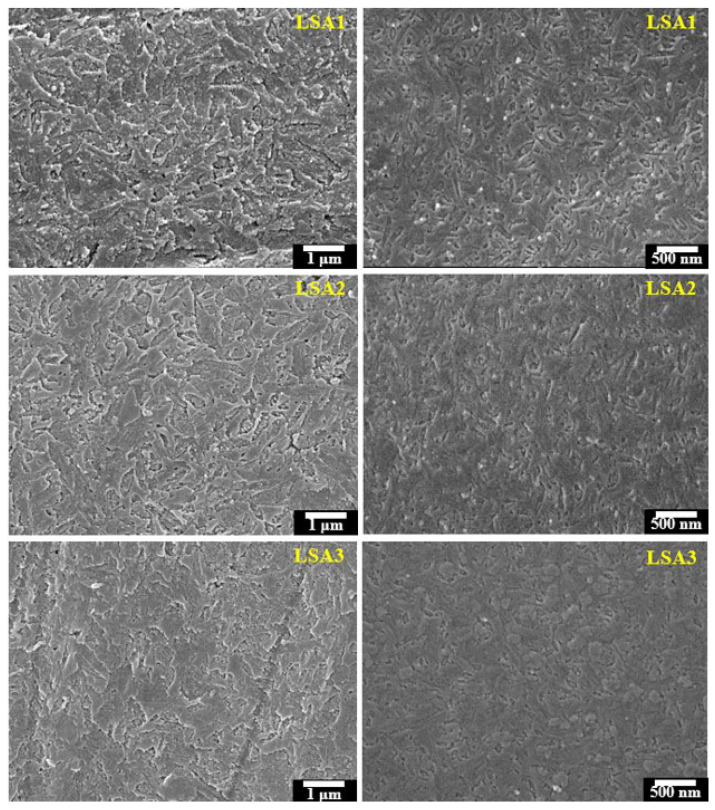
Micrographs of the surfaces (**left**) and fracture of the glass–ceramic samples (**right**) after heat treatment at 600/800 °C.

Figure 3 shows the microstructure of the surface glass–ceramic samples. The results illustrated that the surfaces of the LSA1 and LSA2 samples showed the formation of a rod-like Li_2_Si_2_O_5_ phase that was randomly dispersed. However, the morphology of the crystalline phase of the LSA3 sample was different. With the addition of Al_2_O_3_ to 1.5 mol%, the grain size that was changed to higher increased, and the number of rod-like shapes gradually decreased. Moreover, the shape of the crystals was slightly transformed to the spherical shape of Li_2_SiO_3_, presenting a polymorphic microstructure [26].

These results were confirmed by the fracture surface of the glass–ceramic samples in Figure 3, which shows that in the LSA1 samples, the crystalline shape is rod-like and distributed on the glass matrix of the glass–ceramics. Generally, cracks are always formed at the weaker interfaces of the crystals and propagate through the residual glass matrix. However, in LSA1, the crystal morphology is rod-like. It has a closely packed and multi-directional interlocking microstructure of Li_2_Si_2_O_5_ that deflects and delays crack propagation [27]. Microcracks were formed inside the matrix of glass–ceramics with curves and intersecting paths. This was related to the interlocking structure of Li_2_Si_2_O_5_, which prevented crack propagation in the samples. On the other hand, with increasing Al_2_O_3_ content, the density of Li_2_Si_2_O_5_ crystals decreased and became spherical coexisting with the rod shape in LSA3. Notably, increasing the Al_2_O_3_ content depressed the reaction of Li_2_Si_2_O_5_ but consumed the Li_2_SiO_3_ crystal.

### 3.4. Mechanical Properties

**Figure 4:**
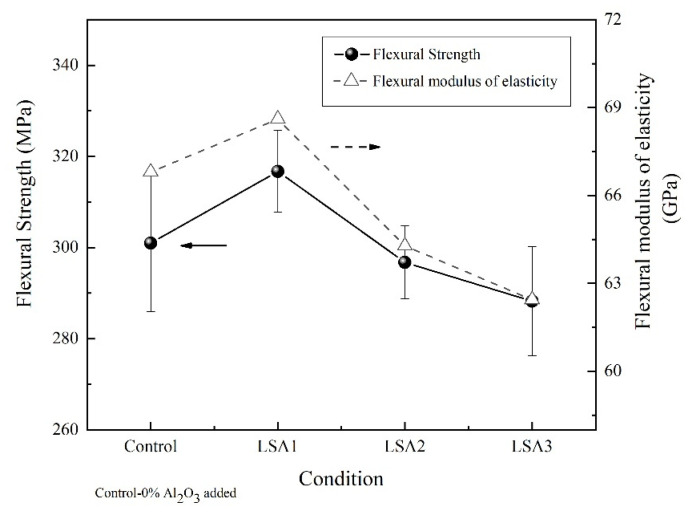
Flexural strength and flexural modulus of elasticity of the glass–ceramic samples with different Al_2_O_3_ contents after heat treatment at 600/800 °C.

**Figure 5:**
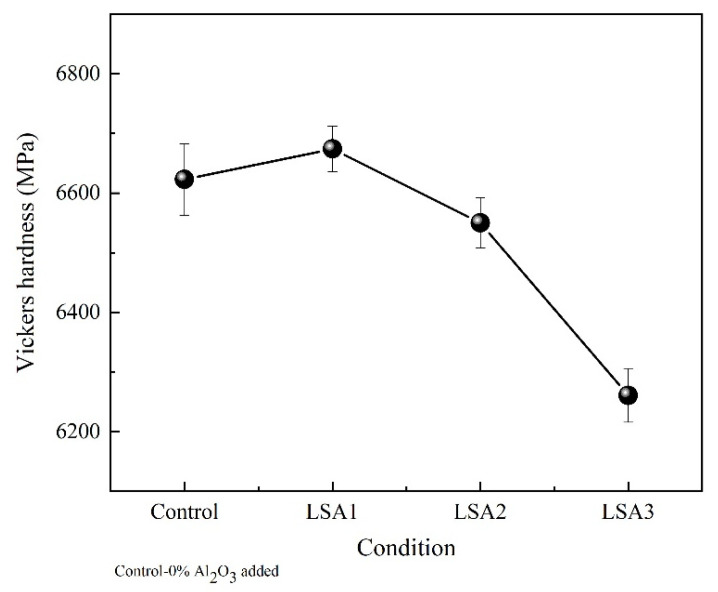
Vickers hardness values of the glass–ceramic samples with different Al_2_O_3_ contents after heat treatment at 600/800 °C.

The improved mechanical properties of glass–ceramics are also a consequence of their higher crystallinity, appropriate morphology of precipitated crystals and low porosity [16,26]. The flexural strength, the flexural modulus of elasticity, and microhardness values of the glass-ceramic samples with different Al_2_O_3_ are shown in Figure 4 and Figure 5. Similar trends can be observed in all the mechanical properties measured in this study with respect to the Al_2_O_3_ contents. The highest mechanical strength values were obtained from the glass–ceramics sample upon adding Al_2_O_3_ to 1.0 mol% and slightly decreased as the Al_2_O_3_ content was increased to 1.5 mol%. A possible explanation is that LSA1 samples contained the highest crystalline phase of rod-like Li_2_Si_2_O_5_, which formed an interlocking microstructure to prevent cracks within the samples. In addition, the increasing Al_2_O_3_ had the effect of decreasing the densification of the crystal, which is related to the amount of glassy phase and its viscosity [28]. In the samples with higher Al_2_O_3_ content, the amount of glassy phase was reduced, and the viscosity of the glassy phase increased, so the addition of a crystalline phase could inhibit densification [28]. This is consistent with the work of Fernandes et al. [29] reporting that the glass sample with the lowest Al_2_O_3_ increased the densification of the crystal which resulted in an improvement in the mechanical properties of lithium disilicate glass-ceramics.

The mechanical strength values of the specimens in the present study were close to those of the commercial lithium disilicate developed by Ivoclar Vivadent company and other studies, as shown in Table 1. Commercial lithium disilicate has a flexural strength and Vickers hardness values of 4400 and 5800 MPa, respectively. These excellent mechanical properties can be attributed to the development of Li_2_Si_2_O_5_ in the sample.

### 3.5. Microshear Bond Strength

The cementation of resin cement for ceramic restorations is a crucial process for clinical success [A1]. Various surface treatments have been used before cementation to improve the bond strength of dental ceramics [34,35]. The micro-shear bond strength test was selected in this study to evaluate the bonding performance owing to its benefits of more uniform stress distribution and lack of damage to specimens that are suitable for the glass–ceramic material [36,37].

In addition to the different mechanical properties, there was no significant difference in μSBS among the three group of samples (LSA1, LSA2 and LSA3) of lithium disilicate glass–ceramics. This might be due to the lower effect of the mechanical properties on the bonding ability between the resin cement and the selected ceramics, as shown in Table 2. However, mechanical properties are crucial and must be considered because ceramics with better mechanical properties provide a longer lifetime for all ceramic restorations [38,39].

The mean μSBS of all the groups with different surface treatments was significantly higher than that of the control group. This result could be attributed to the effectiveness of the chemical bond created by the silane coupling agent and the micromechanical retention created through hydrofluoric acid etching.

Silane promotes the adhesion between the functional methacrylate group of resin cement and the hydroxyl group of the silica-based ceramic. Additionally, a hydrophobic surface can be created by the application of a silane-coupling agent, which allows resin cement to flow [40,41,42].

Hydrofluoric acid can dissolve the glass matrix and increase the surface area for micromechanical retention between resin cement and the etched glass surface [43,44,45], resulting in increased bond strength. However, the lithium disilicate glass–ceramics used in this study contained Al_2_O_3_, which can neutralise the acidity of hydrofluoric acid and impair the etching ability. Moreover, the penetration of resin cement could be interfered with by the insoluble salt formed owing to the interaction between aluminium ions and hydrofluoric acid [46]. This may be the reason for the low μSBS in the group treated with hydrofluoric acid.

The highest μSBS value was obtained by etching with hydrofluoric acid, followed by the application of a silane coupling agent. This method has been recommended as the most effective surface treatment for glass–ceramics [45,47,48,49]. This result might be due to the synergistic combination of mechanical and chemical retention, as the increased surface area created by etching provides more surface area for chemical bonds.

### 3.6. Cellular Activity

**Figure 6:**
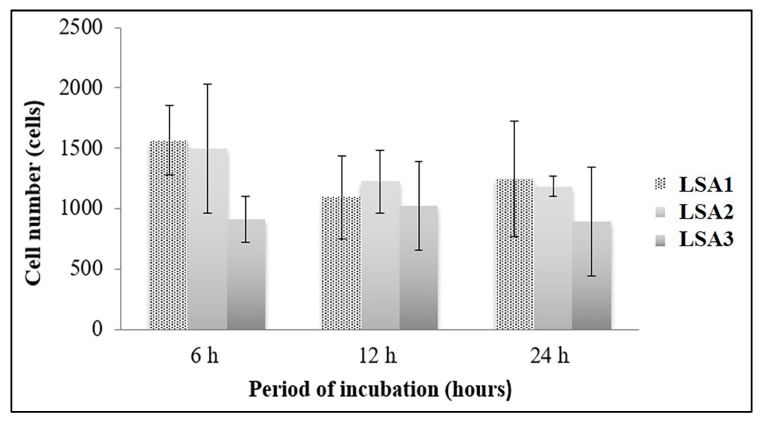
MG-63 cell attachment to lithium disilicate glass-ceramics with various Al_2_O_3_. MTT assay of MG-63 cells attached to lithium disilicate glass-ceramics with various Al_2_O_3_ for 6 h, 12 h, and 24 h. There was no statistical difference among glass-ceramics types and time points.

Although the results showed lower cell numbers attached to LSA3, there was no significant difference among the others (Figure 6). The attachment of the cells onto the surface of the glass-ceramic samples were not affected by the observation period (6–24 h), indicating that the glass-ceramics allowed MG-63 cell attachment, regardless of the concentration of Al_2_O_3_.

**Figure 7:**
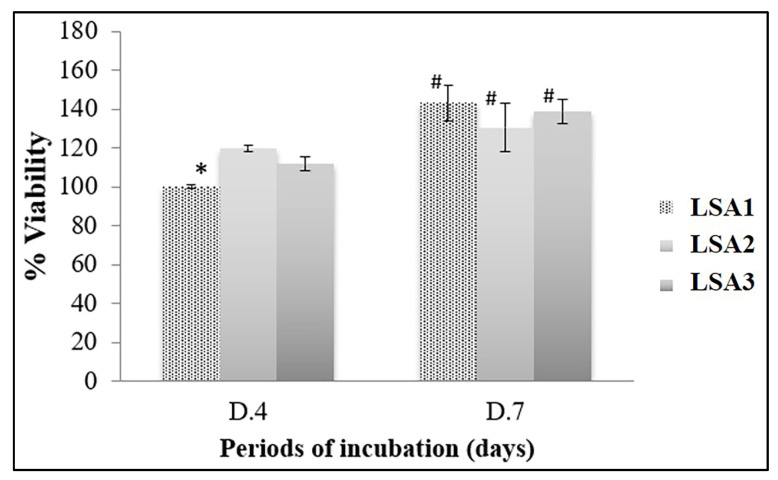
Viability and proliferation of MG-63 cells. Lithium disilicate glass-ceramics with various Al_2_O_3_ were assessed in terms of biocompatibility with MG-63 cells using MTT assay for 4 (D.4) and 7 days (D.7). The results were from a triplicate (*n* = 3) experiment and the statistical difference was tested when *p* < 0.05, which is represented by * (among the material types in each investigation period) and by # (between two investigation periods among the same material types).

Lithium disilicate glass-ceramics with various Al_2_O_3_ were biocompatible with MG-63 cells. Although the MG-63 viability cultured on LSA1 significantly decreased compared with that on LSA2 and LSA3 after 4 days of incubation, this difference was dissipated after 7 days of incubation (Figure 7). The viability of the cells significantly increased over time compared with that at the early time point. This result confirmed the biocompatibility of all types of glasses owing to the ability of MG-63 cell proliferation on all types of glasses.

**Figure 8:**
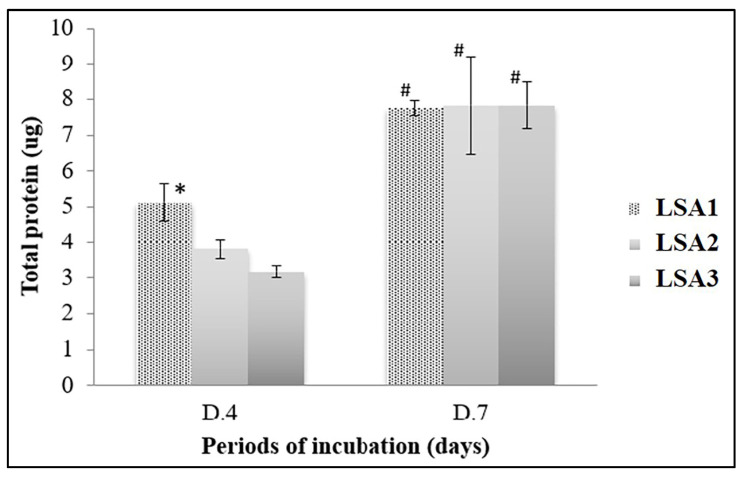
MG-63 protein contents. Total MG-63 protein production after the culture with lithium disilicate glass (LSA3) or lithium disilicate glass supplemented with aluminium at 0.5% mol (LSA1) and with aluminium at 1% mol (LSA2) for 4 (D.4) and 7 days (D.7), as determined with Bradford assay. The results were from a triplicate (*n* = 3) experiment, and the statistical difference was tested when *p* < 0.05, which is represented by * (among the material types in each investigation period) and by # (between two investigation periods among the same material types).

Lithium disilicate glass-ceramic material with various concentrations of Al_2_O_3_-activated protein was produced by the MG-63 cells. The difference in the total protein content was significantly higher in the MG-63 cells cultured with LSA1 after 4 days of culture; however, these contents were similar in all material types after 7 days of culture (Figure 8). When compared between time points, the protein content was significantly increased in all the investigated materials. Taken together, the proliferation results confirmed that the glass specimens, regardless of the presence of aluminium, were biocompatible with MG-63 cells.

**Figure 9:**
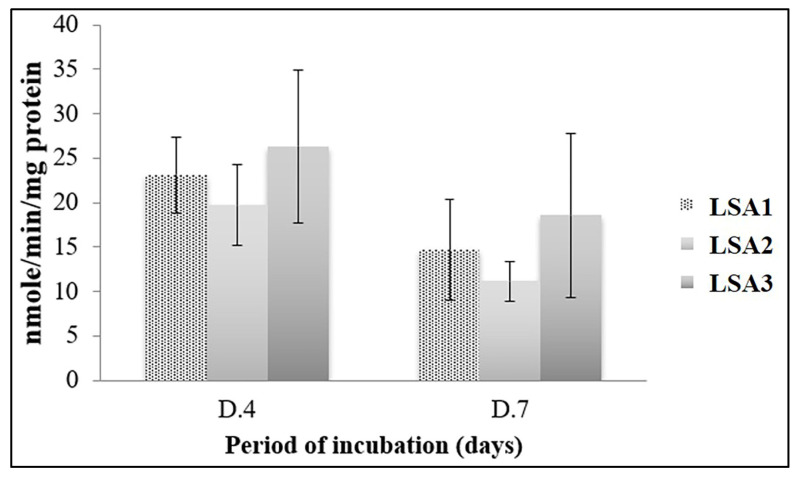
The alkaline phosphatase (ALP) activity. There was no statistical difference among glass-ceramics types and time points.

In terms of the alkaline phosphatase (ALP) activity of MG-63, the MG-63 cells cultured with lithium disilicate glass-ceramics with various Al_2_O_3_ for 4 (D.4) and 7 days (D.7) were assessed for the ALP activity by performing *p*-nitrophenolphosphate conversion assay, and the results are shown in Figure 9. Regarding the expression of a bone formation marker, the ALP activity was not significantly expressed in the MG-63 cells cultured with different types of lithium disilicate glass-ceramics after 4 and 7 days of investigation. Likewise, the ALP activity expression trend was slightly reduced during prolonged investigation periods in all the tested glass-ceramics. This suggests that lithium disilicate glass-ceramics, regardless of Al_2_O_3_, are biologically active, allowing MG-63 cells to normally express the ALP activity, leading to the regulation of bone formation.

**Figure 10:**
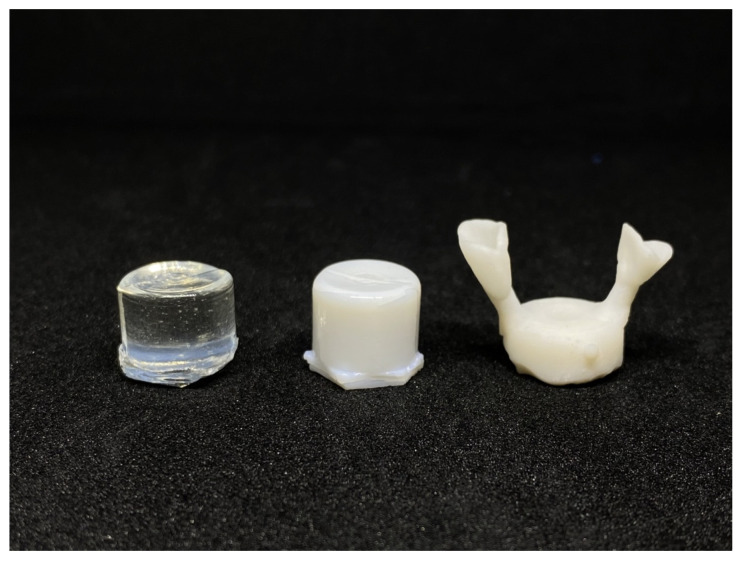
The LSA3 sample before heating (**left**), the LSA3 sample heated at 600/800 °C and the completely pressed object (**right**).

IPS e. max is a lithium disilicate glass–ceramic ingot that delivers high-strength and highly aesthetic materials for press technology. Figure 10 shows LSA3 before and after heating at 600/800 °C. This sample can be pressed into the shape of a dental crown.

## 4. Conclusions

The aim of this study was to investigate the effect of Al_2_O_3_ at the three different contents of 0.5, 1.0 and 1.5 mol% on the mechanical properties of the modified Li_2_O–SiO_2_ glass systems with CeO_2_. The Al_2_O_3_ content played a major role in phase formation changes in glass-ceramic samples. With increasing the content of Al_2_O_3_, the relative content of the Li_2_Si_2_O_5_ phase decreased. The data showed that the LSA1 had the highest crystallinity of Li_2_Si_2_O_5_ as well as the highest mechanical strength correlated with the homogeneous crystallisation of interlocked rod-like Li_2_Si_2_O_5_. In spite of the differences in the mechanical strength, of the samples, all the ceramic conditions showed the same micro-shear bond strength for each surface treatment. An MTT assay was used to test the cellular adhesion and biocompatibility of lithium disilicate glass-ceramic sample for all three contents of Al_2_O_3_. Even though the results were insignificant among the three samples, can be reasonably claimed that these samples are suitable for dental applications.

## Figures and Tables

**Table 1:** Mechanical properties of dental glass-ceramic.

Materials	Commercial Leucite (IPS Empress^®^)	Commercial Leucite (IPS Empress 2)	Commercial Lithium Disilicate	Lithium Disilicate	Lithium Disilicate
Flexural strength (MPa)	90–130	400	400	350–450	316.72
Vickers hardness (MPa)	4001–6500	6500	5800	4001–6500	6685
Ref.	[30,31]	[32]	[28]	[8,33]	This study (LSA1)

**Table 2:** Mean and standard deviation of micro-shear bond strengths.

Surface Treatment	Microshear Bond Strength (Mean ± SD; MPa)		
	LSA1	LSA2	LSA3
No surface treatment (Control)	3.69 ± 0.56 ^a^	3.47 ± 0.58 ^a^	3.23 ± 0.48 ^a^
Silane coating	34.20 ± 2.57 ^c^	37.21 ± 3.43 ^c^	37.97 ± 3.07 ^c^
Hydrofluoric Acid	20.92 ± 2.52 ^b^	20.74 ± 2.85 ^b^	19.34 ± 3.48 ^b^
Hydrofluoric acid + Silane coating	45.34 ± 2.40 ^d^	47.57 ± 4.05 ^d^	47.89 ± 3.10 ^d^

Different superscript letters indicate statistical significance (*p* < 0.05).

## Data Availability

Not applicable.
